# Nutritional Management for Infants with Cystic Fibrosis Born with Meconium Ileus: A 15-Year Review

**DOI:** 10.3390/nu18152463

**Published:** 2026-07-28

**Authors:** Susan Gemma, Anne Rice, Rosara Bass, Mariah Eisner, Katelyn Krivchenia

**Affiliations:** 1 Division of Pulmonary, Sleep Medicine and Cystic Fibrosis, Nationwide Children’s Hospital, Columbus, OH 43205, USA; susan.gemma@nationwidechildrens.org (S.G.); anne.rice@nationwidechildrens.org (A.R.); 2 Division of Gastroenterology, Hepatology and Nutrition, Nationwide Children’s Hospital, Columbus, OH 43205, USA; rosara.bass@nationwidechildrens.org; 3 Department of Pediatrics, The Ohio State University College of Medicine, Columbus, OH 43205, USA; 4 Center for Biostatistics, College of Medicine, The Ohio State University Wexner Medical Center, Columbus, OH 43205, USA; mariah.eisner@nationwidechildrens.org; 5 Biostatistics Resource at Nationwide Children’s Hospital, Abigail Wexner Research Institute at Nationwide Children’s Hospital, Columbus, OH 43205, USA

**Keywords:** meconium ileus, cystic fibrosis, nutrition

## Abstract

**Background/Objectives**: Meconium ileus (MI) related to cystic fibrosis (CF) presents unique nutrition challenges for newborns. The aim of this study was to present a descriptive case series reviewing the management practice and clinical outcomes of infants with CF and MI at a large, quaternary care center. **Methods**: A retrospective analysis examined patients born with CF and MI over fifteen years at a large pediatric hospital in the Midwest. Patient demographics, prenatal imaging data, newborn screening results, stool studies, sweat chloride and details of the clinical course were obtained through the electronic medical record (EMR). Outcomes including length of stay (LOS), timing of surgery, and duration of total parenteral nutrition (TPN) needs were compared between infants with and without a surgical ostomy. Associations of MI severity with CF genotype, stool elastase, and prenatal imaging findings were evaluated. **Results**: A total of 30 neonates (21 female, 9 male) were included. Surgery was required in 26 infants, with 18 requiring ostomy placement. There were no significant correlations between CF genotype, stool elastase, or prenatal imaging findings with need for ostomy placement. Compared to those without ostomy placed, infants with ostomies had significantly longer median [IQR] LOS (71 [61, 105] vs. 22 [16, 32] days; difference 49, 95% CI 34–77) and duration of TPN (52 [41, 74] vs. 8 [6, 12] days; difference 43, 95% CI 25–62) (*p* < 0.001 for both). **Conclusions**: This work highlights the complex medical needs of these infants, requiring prolonged hospitalization and TPN for adequate nutrition. We share our institution’s approach to the nutritional management infants with CF and MI, with a focus on collaboration needed with the multidisciplinary team.

## 1. Introduction

Cystic fibrosis (CF) is an autosomal recessive disease resulting from a defect in the cystic fibrosis transmembrane conductance regulator (*CFTR*) gene, affecting one in approximately 3000 live births in the United States. The CFTR protein facilitates the transport of sodium, chloride and water across cellular membranes. Dysfunction of CFTR alters the sodium/chloride content on the surface of epithelial membranes, leading to abnormally thick, sticky secretions, especially in the respiratory and gastrointestinal tracts.

Meconium ileus (MI) is the earliest possible manifestation of CF and is often diagnosed within hours after birth, with suggestive findings visible on prenatal ultrasound [1]. Meconium is the first stool passed by the newborn, usually within 24 to 48 h of life. In infants with CF, an increased accumulation of thick mucus lining the gastrointestinal tract may result in failure of the meconium to pass, and bowel obstruction can occur. Approximately 10–20% of infants with CF are born with MI, with up to 75% requiring surgical intervention [1,2,3,4,5]. Infants with MI experience symptoms of small bowel obstruction, including abdominal distention and bilious emesis as well as failure to pass meconium within the first one to two days of life.

### 1.1. Pathophysiology of Meconium Ileus

Class I through III *CFTR* mutations (including F508del, G542X, W1282X, R553X and G551D) are most commonly associated with MI and pancreatic insufficiency [2]. CFTR is responsible for the secretion and transport of chloride, bicarbonate, and fluid within the small intestine [2]. Mucus is composed of glycoproteins (mucins) which function to lubricate and protect the epithelium of the GI tract. In healthy individuals, the mucus layer in the small intestine is loose and movable. Dysfunctional CFTR results in reduced bicarbonate, causing decreased luminal pH. An acidic and dehydrated milieu is created in the small intestine, contributing to an accumulation of thick, viscous mucus that is described as “stagnant” [2,6,7]. This abnormally dense mucus is a contributing factor to the formation of the adherent, viscous meconium in the small intestine that eventually leads to a physical obstruction in the terminal ileum. While animal studies have suggested a role for pancreatic enzymes in digestion of meconium [8], the co-occurrence of MI and exocrine pancreatic insufficiency is likely multifactorial, with more severe alterations in mucous viscosity occurring in more severe genotypes (the same which would also be associated with decreased CFTR function in the pancreas and thus associated with PI).

### 1.2. Diagnosis, Classification and Management of MI

While most newborns in the United States with CF are identified by state-specific newborn screening, this process relies on elevated serum immunoreactive trypsinogen (IRT) levels as a marker of pancreatic injury in the infant’s blood. IRT levels of infants with MI may be falsely low, making the newborn screen less reliable for infants with MI [9,10]. Confirmatory genetic testing of *CFTR* is often required for CF diagnosis since these infants may be too small and malnourished to permit accurate sweat chloride testing [2]. MI related to CF can also be suspected when findings of hyperechoic bowel or meconium peritonitis are visualized on prenatal ultrasound, though meconium-related obstruction may also occur in non-CF disorders [2,11].

When a newborn presents with abdominal distention and/or bilious emesis after initiation of feeds, evaluation for obstructive processes, including MI, begins with an abdominal radiograph which may reveal dilated loops of the small bowel [1]. MI is classified as “simple” or “complex/complicated.” Simple MI is defined as an obstruction with thick, inspissated meconium in the small intestine. Complex or complicated MI involves an obstruction of the intestine resulting in complications such as prenatal volvulus, ischemic necrosis, intestinal atresia or perforation of the bowel, necessitating a surgical emergency [1,2]. Surgical intervention may result in a resection of the intestine.

In simple MI, use of a hyperosmolar enema may successfully dislodge obstructing meconium in 30–50% of cases [2,3]. If attempts to alleviate the obstruction are unsuccessful or are complicated by bowel perforation, the infant will require emergent surgical intervention, which may require a bowel resection depending upon the extent of meconium burden and presence of related complications. The type of surgical intervention performed will impact the post-operative course [3]. In many cases, a primary anastomosis is not feasible, and a temporary ostomy and/or mucous fistula is created [1,3].

Any injury to an infant’s small intestine can greatly compromise nutrient absorption, placing the infant at risk for malnutrition. The nutritional management of infants with CF and MI can be very challenging, and while this is acknowledged in review articles and by the CF dietitian community, there are no evidence-based guidelines pertaining to the nutritional management of infants recovering from treatment of MI.

We present a descriptive case series and single-center experience of nutritional management of 30 infants with CF and MI born over a 15-year period. The primary objective of this work is to describe both the management practices and clinical outcomes of our cohort and to retrospectively identify factors that may contribute to hospital lengths of stay (LOS). We hypothesized that infants who required surgical ostomy placement would have longer LOS than infants with MI who did not require an ostomy.

## 2. Materials and Methods

This was a single-center retrospective chart review approved by the institution’s IRB (STUDY00001800). There are typically 10–15 new diagnoses of CF at our center each year. Patients were included if they were born between 2010 and 2024 and cared for at our large quaternary care center with a diagnosis of MI and CF. Patients were identified using diagnosis codes for MI and CF and by subsequent EMR review. The diagnosis of MI was confirmed by individual review of operative reports and documentation from the NICU and surgery teams. The MI was classified as “complicated” if the obstruction resulted in ischemic necrosis, volvulus, or bowel perforation. The diagnosis of CF was confirmed by newborn screen, *CFTR* genetic testing with two disease-causing variants, and/or sweat chloride testing >60 mmol/L, depending on the clinical scenario. No infants with both CF and MI were excluded. When available on the EMR, prenatal ultrasound (US) and magnetic resonance imaging (MRI) reports were reviewed to identify possible signs of MI (including echogenic bowel, small bowel dilation, or bowel atresia). Our institution serves as the surgical referral center for several outlying NICU and newborn nurseries in Ohio. Clinically, patients with CF and MI at our center are managed by NICU specialists with consultations from pediatric surgery and pulmonary medicine. The physician teams turn-over every 1–3 weeks, depending on the specialty. Four dedicated CF-specific dietitians are actively involved in the nutritional management of all infants with MI and CF throughout their hospitalization.

Patient demographics, newborn screening results, stool studies, sweat chloride and clinical course were obtained from EMR review. Stool elastase values below the limit of detection were imputed to the limit so that stool elastase could be analyzed continuously. Variables were visualized with bar or violin/box plots to check for outliers and distributional assumptions. Patient demographics and clinical characteristics were summarized using frequency (percentage) for categorical variables, mean (standard deviation [SD]) for normally distributed continuous variables, and median (interquartile range [IQR]) for skewed continuous variables, overall and by need for ostomy. Outcomes and management including length of stay (LOS), days of life (DOL) at surgery, DOL feeds started, and duration of total parenteral nutrition (TPN) were compared by presence of ostomy using the Wilcoxon rank sum test with estimator for difference of location parameters (median of the difference between a sample from the first group and a sample from the second group) and 95% confidence interval (CI) or two sample *t*-test with differences in mean and 95% CI. Associations with CF genotype, stool elastase, positive prenatal imaging findings, and LOS were evaluated for MI severity (simple vs. complex), need for ostomy, and fistula refeeding with Fisher’s exact, Wilcoxon rank sum, or *t*-tests. Each test was performed on patients with available data for the variables of interest, and sample size was reported in the presence of missing data. Normality was assessed through variable visualizations. Two-sided *p*-values < 0.05 were considered statistically significant; *p*-values were not adjusted due to the exploratory nature of the study. All statistical analyses were performed in R version 4.4.2 (R Core Team, Vienna, Austria) with reproducible programming in Quarto.

## 3. Results

A total of 30 neonates (21 female, 9 male) with MI due to CF were included in this analysis. Patient characteristics are presented in Table 1. The majority (73%) of infants were born at term (≥37 wks gestation). IRT levels on the newborn screen ranged from 25 to 234 ng/mL, with mean of 120 ng/mL (SD 53). Sweat chloride testing was completed in 28 of the 30 infants at the time of data review, with a range of 79–107 mmol/L and mean of 95 mmol/L (SD 6). All infants were found to be pancreatic insufficient, with collected stool elastase values (measured after re-anastomosis) ranging from <10–171 µg/g.

**Figure 1:**
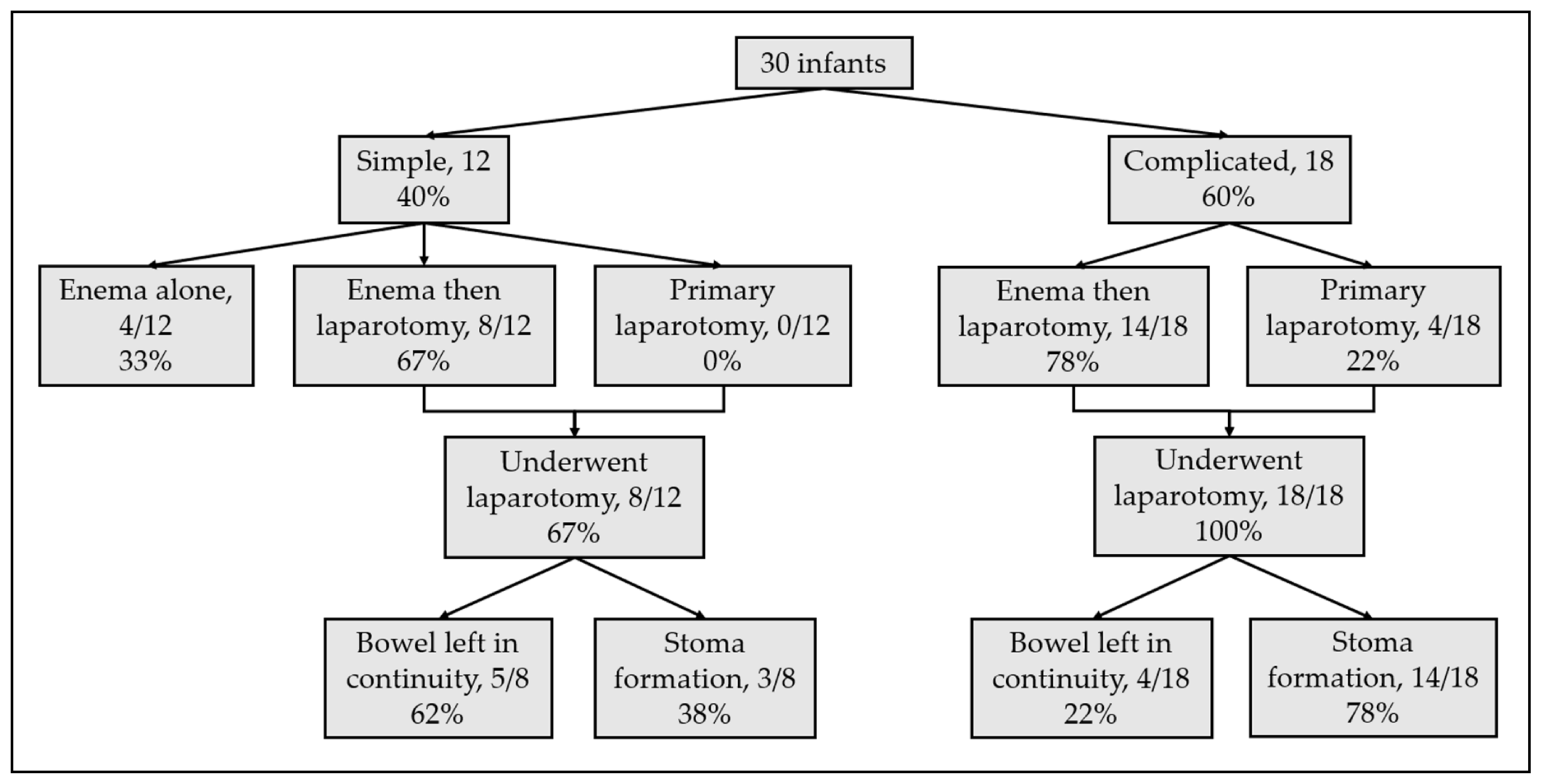
Classification and surgical management of MI.

Figure 1 describes the initial MI classification (simple vs. complicated) and management of these infants, including whether infants ended up requiring laparotomy and whether or not stoma formation was required. Two infants were classified as having short bowel syndrome with <100–120 cm small bowel remaining.

Associations of genotype, prenatal imaging findings, and stool elastase measures with need for surgical ostomy were explored and are shown in Table 2, with no statistically significant differences between groups. Prenatal imaging reports were available for only 13 infants in our cohort, with 11 having signs of possible MI.

Table 3 displays data related to LOS, timing of surgical intervention (when required), timing of initiating enteral feeds, and duration of TPN for these infants. For the 18 infants who required ostomy, surgical re-anastomosis occurred at a median of 49 days of life (IQR 42, 58). Ninety percent (27/30) of infants were started on expressed breast milk (EBM) when feeds were initiated. Additional calories through fortification of EBM or formula were required in 80% (24/30) of our cohort at some point during their initial hospitalization. Of these 18 infants, eight were re-fed ostomy output via the mucous fistula. When compared to those who did not receive re-feeding, there was no significant difference in LOS (median of 88 days [IQR 65, 122] vs. 66 days [56, 93], difference = 23 days, 95% CI = −9 to 73, *p* = 0.2) or duration of TPN (median of 64 days [IQR 52, 86] vs. 45 days [20, 67], difference = 21 days, 95% CI = −1 to 47, *p* = 0.068). Seventy percent of the infants (21/30) in our cohort were transferred from the NICU to a dedicated pulmonary service on the general hospital floor prior to discharge, while nine infants (30%) were directly discharged from the NICU.

## 4. Discussion

### 4.1. Clinical Determinants of Outcomes for MI

To our knowledge, this is the largest series describing clinical outcomes of infants with CF and MI in the United States reported to date.

Given the need to balance introduction of enteral nutrition with ostomy output and surgical management, it is not surprising that our data demonstrated significantly longer duration of TPN use and overall LOS for infants who required ostomy compared to those without any operative intervention, or those with an operative intervention that was amenable to primary anastomosis. The longer LOS and TPN duration likely reflect the overall disease severity at presentation. In a cohort of 26 patients with CF and MI in France, Munck et al. reported the need for nutritional support in all surgical patients (with or without intestinal resection), with a much higher mean TPN duration of 97 days [12]. Variability between cohorts may reflect both differences in patient populations examined as well as non-patient factors including availability of TPN and provider practice across institutions.

Our population differs from French [12] and British [5] CF/MI cohorts previously reported. The majority of our cohort had homozygous F508del genetics, while Munck et al. reported having an equal proportion of infants with homozygous and heterozygous F508del variants [12]. Our cohort’s median birth weight was similar to both other reported cohorts. There was a notable female predominance in our population compared with the close to even male:female distribution reported elsewhere [5,12]. It is unclear why this may be as all infants with CF and MI were included in our cohort without regards to biologic sex. To our knowledge there are no known referral biases related to this population at our center.

The major limitation of the data we present is related to the retrospective, single-center design which limits the generalizability of the results. The infants in our cohort were managed by a dedicated team of CF-specialized dietitians who developed their approach over time and therefore made decisions based on their clinical experience where published data was lacking. Because of this small sample size, we did not exclude infants with other comorbid conditions (i.e., prematurity, bronchopulmonary dysplasia, etc.) and could not correct for these comorbidities in analyses for LOS and TPN usage. Additionally, the small sample size limits our ability to draw firm conclusions from analyses performed (particularly regarding interpretation of negative findings in Table 2). Conclusions about impact of clinical interventions on health outcomes and resource usage are difficult to ascertain. Because of these limitations, the data we present are descriptive and do not prove causation.

Despite these limitations, we present our center’s experience with CF-related MI to serve the broader dietitian community. While MI is a known severe presentation for neonates with CF, it occurs so rarely that many small and medium-sized NICU teams do not see these babies frequently enough to feel confident in the nuances of caring for this population. Our dietitian team is regularly contacted to discuss nutritional management of these infants given the lack of published guidance.

### 4.2. Our Center’s Clinical Practice

**Figure 2:**
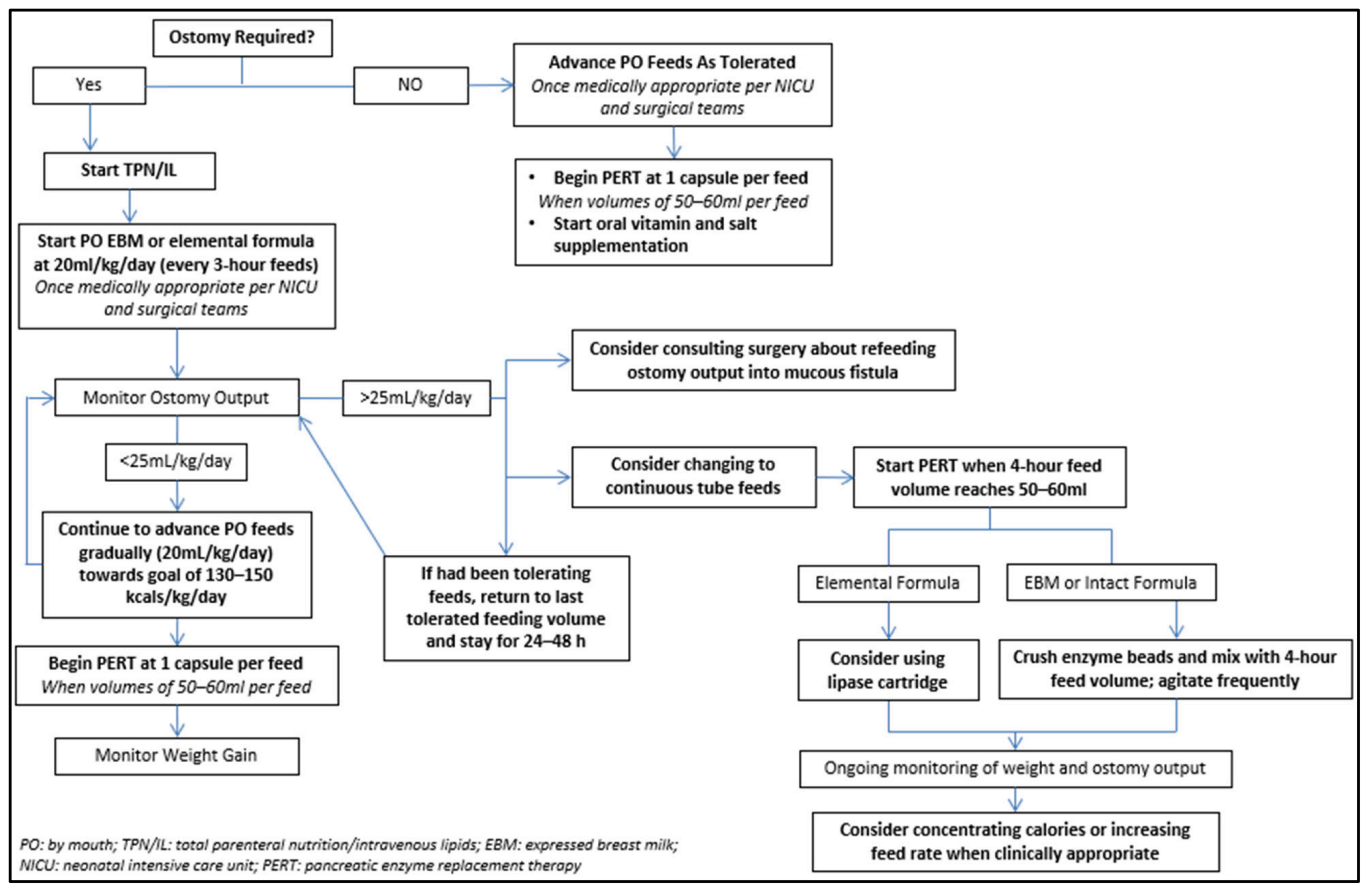
Center-specific algorithm for nutritional management of meconium ileus and cystic fibrosis.

Working with a dedicated multi-disciplinary team, we developed a stepwise approach to nutritional management in MI related to CF. Figure 2 depicts a simplified version of the way our center managed nutrition in these infants. This is a general representation of our approach, and not all infants in our cohort had care following every step of this algorithm. The following sections describe important aspects of nutritional support for this population. As no published guidelines exist specifically for the nutrition in CF with MI, we describe the published literature supporting our practice but recognize that our approach is center-specific and expert-driven. Figure 2 is meant to provide an algorithm that clinical teams may follow if they agree with the reasoning of our team and supporting evidence provided.

### 4.3. Management of Feeds

Many factors determined the nutrition management and progress for infants following MI. Initiation of oral or enteral feeds was dependent on whether surgery was required, the location of bowel resection, and how much intestine was removed. If the obstruction was resolved with hyperosmolar enemas and no surgical intervention, oral feeds were initiated with human milk or formula, and advancement to full feeds was generally well tolerated. For patients with intestinal resection including an ostomy (with or without mucous fistula), full parenteral nutrition was given until advancement of oral feeding was recommended by the surgical team. Oral or enteral feeds were cautiously advanced with expressed breast milk or formula. In many cases, TPN and intravenous lipids (IL) in combination with oral or enteral feeds were needed to provide adequate calories until re-anastomosis of the bowel. There is no definite recommendation for feed advancement in these infants post-operatively, with reported rates varying widely [13]. Our approach for infants is to target a volume advance of 20 mL/kg/day, monitoring closely for tolerance.

Indications of poor tolerance include elevated stoma output, emesis, and poor weight gain [13]. High-ostomy output can result in malabsorption and intestinal failure, with various definitions of high output ranging from >20–50 mL/kg/day [13,14]. At our center, oral or enteral feeds were gradually advanced when ostomy output was less than 25 mL/kg/day and remained stable. In some cases, enteral feeds were better tolerated than oral feeds due to ability to administer feeds at a slower and more consistent rate. To maintain oral feeding skills, infants were allowed a daily pause in enteral feeds for 1–2 h to permit oral intake.

There is evidence to support refeeding the ostomy contents through the mucus fistula to promote peristalsis, mucosal growth, and facilitate feed tolerance once enteral or oral feeds have been restarted after reanastomosis [15], without surgical complications [16]. Mucous fistula refeeding has been associated with fewer days of TPN, faster time to the goal of full feeds, and shorter hospital stay secondary to maintaining distal bowel function [15,16,17]. When we compared infants who were refed using this technique to those who were not, we did not find a statistically significant difference in LOS or duration of TPN. However, we are likely underpowered to appreciate significant changes, and at our institution, the decision to refeed via the ostomy was surgeon-dependent, thus subjecting results to multiple other confounding factors, as would be expected based upon the retrospective nature of the study.

### 4.4. Pancreatic Enzyme Therapy

Timing of initiation of pancreatic enzymes is an important consideration for infants with MI, and it is possible that increased ostomy or poor weight gain may be attributable to lack of early enzyme replacement therapy. There are no published guidelines addressing the ideal time to start PERT in hospitalized infants taking small volumes enterally. In clinical practice, dosing of pancreatic enzymes is typically determined by patient weight or based on fat grams in the volume of human milk or formula. Prescribing information for commercially available enzymes provides dosing based on 120 mL of formula [18,19,20,21]. CF nutritional experts cite a starting volume of ≥60 mL for PERT supplementation [22].

As small volumes of human milk or formula were initiated in our NICU population, the approach of our center has been to consider dosing based on the amount of fat being given. Small volumes of human milk or formula contain a negligible amount of fat, with 5 mL of human milk or standard formula providing approximately 0.2 g fat and 30 mL providing only 1.2 g fat. Our practice was to start enzyme replacement when the volume of each individual feed was between 50 and 60 mL. Because of the small volumes of fat at the onset of enteral feeds in this population, an increase in ostomy output is not likely due to the absence of pancreatic enzyme therapy. Other factors should be considered, including the rate of feeds and length of residual bowel, as decreased bowel length may result in decreased absorptive capacity and higher ostomy output [13,23].

**Figure 3:**
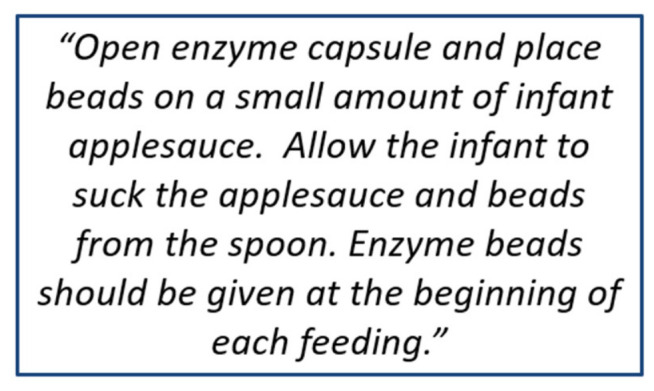
Example text for enzyme order.

Once the infant was ready to start pancreatic enzyme therapy, the family and bedside NICU nurse were taught how to administer enzyme beads. The CF dietitian reviewed rationale for initiating pancreatic enzymes and the use of applesauce as a vehicle for administering the enzyme beads. The volume of applesauce on the spoon with the enzyme beads was kept to a minimum as the hyper-osmolarity of the fruit puree can contribute to frequent stools. This process was clearly documented in the medical record, so all care providers had access to this education. Figure 3 provides an example of text added to the enzyme order.

For infants unable to take oral feeds, crushed beads from a low-strength, enteric-coated enzyme capsule were added to the tube feeding bag. It may be necessary to gently agitate the feeding bag to help the crushed enzymes to mix with the formula or EBM. This method allows for enzyme administration in the smallest of infants to ensure intestinal absorption when feeding volumes are appropriate. A commercially available enzyme cartridge can also be used in line with tubing for enteral feeding. This cartridge contains lipase to facilitate the hydrolysis of fat in formulas, and compatibility charts are available for reference [24]. Generally, our practice has been to use this immobilized lipase cartridge in older infants and children who required continuous enteral feeds.

The CF dietitians followed up frequently with the patient and family throughout admission to observe how the enzyme beads were being given and to assess the tolerance of enzymes and the feeding regimen. Ongoing education was sometimes necessary based on interactions with family or NICU staff. As the volume of feeds was advanced, enzyme dosing was closely monitored and adjustments made as clinically indicated. Daily average weight gain and stool output were additional factors that may have led to adjustment of the enzyme dose.

### 4.5. Calorie Requirements

Infants with MI have increased calorie requirements secondary to fat malabsorption, the need for catch-up growth, and post-operative healing. The recommendations for energy needs for infants with CF range from 110 to 200% above the recommended calorie intake for non-CF infants [25,26]. Reaching this calorie goal is challenging given the likelihood of intolerance when feeding volumes are quickly increased post-operatively. Thus, as the newborns were gradually advanced with enteral or oral feeds, TPN/IL were used to help meet caloric needs. We found that aiming for a total goal of 130–150 kcal/kg/day tended to support desired growth outcomes in these infants, though this is based on clinical experience, and formal study has not been performed using this caloric intake goal. Some infants may reach growth goals with lower kcal/kg/day, highlighting the importance of ongoing monitoring and feed adjustment based on growth and ostomy output.

If human milk is available, this is always the preferred source of nutrition. The decision to introduce a standard, semi-elemental or elemental formula was dependent on the degree of intestinal surgery, location of the ostomy, as well as tolerance of oral intake. For infants requiring intestinal resection with an ostomy, the volume of enteral or oral feeds may have been kept stable if there was increased ostomy output or if the infant exhibited poor oral intake. It was often necessary to concentrate formula or fortify expressed breast milk to help meet energy needs. Increasing the caloric density of feeds was done with a gradual and stepwise approach given the risk of worsening ostomy output related to increased osmolality.

### 4.6. Vitamin and Salt Supplementation

When TPN was discontinued, CF-specific vitamin drops were initiated. The standard dose for these vitamins is 0.5–1 mL/day [26], but infants may have better tolerance when the dose is divided to 0.25–0.5 mL twice a day. The CF dietitian included in the nutrition recommendations that vitamins should be given at the same time as a feeding and enzymes to promote optimal absorption of the vitamin components.

The standard salt supplement for a newborn with CF is 1/8 teaspoon per day [26]. In the hospital setting, the sodium chloride solution dose of 12.5 mEq per day was ordered through the pharmacy. This dose was divided into 4 mEq three times per day and was equivalent to 1/8 teaspoon salt. It was important to ensure that this was converted back to table salt supplementation at time of discharge for ease of management at home.

Oral medication suspensions can contribute to an increased stool output due to a hyperosmolar solution. Recently added medications were considered if ostomy or stool output increased unexpectedly.

### 4.7. Ongoing Monitoring

Although laboratory monitoring for maintenance and adjustment of TPN is generally managed by the NICU team, the CF dietitian collaborated with the NICU dietitian to closely monitor the infant’s weight and stool or ostomy output. The patient’s length and head circumference were measured weekly. The CF dietitian also assessed daily calorie and protein intake, especially as the infant was weaned from TPN/IL and as oral or enteral feeds were advanced. Our practice was for the CF team to participate in the NICU team patient rounds at least weekly.

Chronic sodium deficiency is associated with poorer weight gain, and having an ostomy increases the risk of higher salt losses [26]. If an infant demonstrates sub-optimal weight gain despite adequate caloric intake and without a significant increase in ostomy output, urine sodium was obtained. This was only required in seven of the cases we presented, with two infants requiring supplementation using a target urine sodium of 20–120 mmol/L [27]. If urine sodium was sub-optimal, increased sodium supplementation was given and urine sodium continued was trended to ensure improvement.

It is essential to note that pancreatic stool elastase testing is not appropriate for infants with an ostomy in place. Stool elastase is measured as a concentration and thus is not reliable from liquid stool contents, such as an ostomy, and could be falsely low [2,28]. As many infants with MI have both CF and pancreatic insufficiency, our practice was to presume pancreatic insufficiency in these infants with CF until proper confirmatory testing could be completed.

Promoting positive and steady weight gain in these medically complex neonates can be a significant challenge. While our standard expectation for weight gain in infants is 30 g/day, this may not be a realistic goal until re-anastomosis of the newborn intestine, especially if feeds, ostomy output and urine sodium levels have been optimized. While infants without CF who undergo ileostomy placement may be able to be discharged home before surgical re-anastomosis, the significant challenges in attaining adequate growth in this population made this inadvisable for infants with CF at our center. While this may not be feasible in all healthcare systems, our practice was for infants to remain hospitalized from the time of initial presentation through re-anastomosis due to the need for close monitoring of the infant’s weight, ostomy output and tolerance to oral feeds.

Ongoing collaboration between the NICU, surgery and CF teams was paramount to care of this population. In the midst of medical team turnover during hospitalization, the CF dietitian team served as a means of consistent communication with families regarding nutrition and growth. Other important teams involved in their care included Occupational Therapy (to assess oral feeding skills), Lactation (to encourage, support and guide an often very overwhelmed mother in the strategies of breastfeeding), and Social Work (to navigate the complexities of health insurance and other financial programs). Our center is fortunate to have an Intestinal Rehab Service to help manage infants who meet criteria for short bowel syndrome after surgical intervention.

### 4.8. Discharging Home

We believe infants with CF and MI at our institution benefited from transferring out of the NICU to a specialized pulmonary unit (or general medicine unit with pulmonary consultation) prior to discharge. This allowed for ongoing CF education and close monitoring of weight and stool patterns. Our CF team ensured appropriate weight trajectory on home feeding regimen prior to discharge (ideally with three consecutive days of appropriate weight gain). The CF dietitian provided home-going education on pancreatic enzyme dosing, CF-specific vitamins, salt supplementation and feeding regimen. They reviewed the infant’s eligibility for the Women, Infants, and Children (WIC) program and assisted parents in making arrangements with their local branch. This ensured no gap in formula or supplemental nutrition for nursing mothers.

## 5. Conclusions

We present 15 years’ experience of our center’s nutritional management of infants with CF who presented with MI. This work highlights the complex medical need of these infants, requiring prolonged hospital LOS and TPN/IL use for adequate nutrition. With a lack of standardized guidelines for care of this population, we present our clinical approach to providing nutrition to these infants. Multi-centered, prospective studies on impact of various surgical techniques or post-operative management could be meaningful to the future outcomes of this delicate patient population.

## Figures and Tables

**Table 1:** Patient characteristics.

Characteristic ^1^	N = 30 ^2^
Sex	
Female	21 (70.0%)
Male	9 (30.0%)
Race	
White	28 (93.3%)
Non-white	2 (6.7%)
Birthweight (g)	3001 (580)
Term birth	
No (<37 weeks)	8 (26.7%)
Yes (≥37 weeks)	22 (73.3%)
Gestational age (wk)	38.4 (36.9, 39.3)
Genotype	
Homozygous F508del	22 (73.3%)
Heterozygous F508del	6 (20.0%)
Other	2 (6.7%)
Meconium ileus severity	
Complex	18 (60.0%)
Simple	12 (40.0%)
Ostomy	18 (60.0%)
Stool elastase (µg/g)	15 (10, 22)

^1^ N = 30 for all variables. ^2^ n (%); Mean (SD); Median (Q1, Q3). Abbreviation: SD = standard deviation.

**Table 2:** Associations between patient characteristics and need for surgical ostomy.

Characteristic	N	Ostomy N = 18 ^1^	No Ostomy N = 12 ^1^	Difference ^2^	95% CI ^2^	*p*-Value ^2^
Genotype	30			−31%	−65%, 3.8%	0.2
Homozygous F508del		11 (61.1%)	11 (91.7%)			
Heterozygous F508del or other		7 (38.9%)	1 (8.3%)			
Prenatal imaging diagnosis ^3^	13			−25%	−71%, 21%	0.7
Yes		6 (75.0%)	5 (100.0%)			
No		2 (25.0%)	0 (0%)			
Stool elastase (µg/g) ^4^	29	15 (10, 16)	16 (13, 29)	−1.0	−12, 1.0	0.3

^1^ n (%); Median (Q1, Q3). ^2^ 2-sample test for equality of proportions with continuity correction; Wilcoxon rank sum test. ^3^ Presence of echogenic bowel or bowel dilation indicating MI by fetal US or MRI. ^4^ Values below the limit of detection were imputed to the lower limit. Abbreviation: CI = confidence interval, MI = meconium ileus, MRI = magnetic resonance imaging, US = ultrasound.

**Table 3:** Outcomes and management related to meconium ileus, overall and by need for surgical ostomy.

Characteristic	N	Overall N = 30 ^1^	Ostomy N = 18 ^1^	No Ostomy N = 12 ^1^	Difference ^2^	95% CI ^2^	*p*-Value ^2^
Length of stay (d)	30	58.5 (23.0, 93.0)	71.0 (61.0, 105.0)	21.5 (16.0, 32.0)	49	34, 77	**<0.001**
DOL at surgery (d)	26	2.0 (1.0, 4.0)	2.0 (1.0, 2.0)	3.0 (2.0, 7.0)	−2.0	−5.0, 0.01	**0.020**
DOL feeds started (d)	30	10.4 (3.9)	10.7 (3.9)	9.8 (4.1)	0.89	−2.2, 4.0	0.6
Duration of TPN (d)	30	36.5 (8.0, 67.0)	51.5 (41.0, 74.0)	7.5 (6.0, 12.0)	43	25, 62	**<0.001**

^1^ Median (Q1, Q3); Mean (SD). ^2^ Wilcoxon rank sum test; Welch Two Sample *t*-test. Abbreviation: CI = confidence interval, DOL = days of life, SD = standard deviation, TPN = total parenteral nutrition.

## Data Availability

Study data is being maintained in accordance with our institution’s IRB. Please reach out to the corresponding author for requests regarding this data.

## Abbreviations

The following abbreviations are used in this manuscript:

CF	Cystic fibrosis
CFTR	Cystic fibrosis transmembrane conductance regulator
MI	Meconium ileus
GI	Gastrointestinal
IRT	Immunoreactive trypsinogen
NICU	Neonatal intensive care unit
IRB	Institutional review board
EMR	Electronic medical record
SD	Standard deviation
IQR	Interquartile range
LOS	Length of stay
DOL	Day of life
TPN	Total parenteral nutrition
IL	Intravenous lipids
EBM	Expressed breast milk
FDA	Food and Drug Administration
mEq	Milliequivalent
WIC	Women, Infants, and Children

## References

[1] Tobias J., Tillotson M., Maloney L., Fiakowski E. (2022). Meconium ileus, distal intestinal obstruction syndrome, and other gastrointestinal pathology in the cystic fibrosis patient. Surg. Clin. N. Am..

[2] Sathe M., Houwen R. (2017). Meconium ileus in cystic fibrosis. J. Cyst. Fibros..

[3] Carlyle B., Borowitz D., Glick P. (2012). A review of pathophysiology and management of fetuses and neonates with meconium ileus for the pediatric surgeon. J. Pediatr. Surg..

[4] De Lisle R., Borowitz D. (2013). The cystic fibrosis intestine. Cold Spring Harb. Perspect. Med..

[5] Long A.M., Jones I., Knight M., McNally J. (2021). Early management of meconium ileus in infants with cystic fibrosis: A prospective population cohort study. J. Pediatr. Surg..

[6] Gustafsson J.K., Ermund A., Ambort D., Johansson M.E., Nilsson H.E., Thorell K., Hebert H., Sjövall H., Hansson G.C. (2012). Bicarbonate and functional CFTR channel are required for proper mucin secretion and link cystic fibrosis with its mucus phenotype. J. Exp. Med..

[7] Johansson M., Sjovall H., Hansson G. (2013). The gastrointestinal mucus system in health and disease. Nat. Rev. Gastroenterol. Hepatol..

[8] Nambiar G.G., Szachowicz S.G., Zirbes C.F., Hill J.J., Powers L.S., Meyerholz D.K., Thornell I.M., Stoltz D.A., Fischer A.J. (2024). Pancreatic enzymes digest obstructive meconium from cystic fibrosis pig intestines. Front Pediatr..

[9] Rusakow L.S., Abman S.H., Sokol R.J., Seltzer W., Hammond K.B., Accurso F.J. (1993). Immunoreactive trypsinogen levels in infants with cystic fibrosis complicated by meconium ileus. Screening.

[10] Sontag M.K., Corey M., Hokanson J.E., Marshall J.A., Sommer S.S., Zerbe G.O., Accurso F.J. (2006). Genetic and physiologic correlates of longitudinal immunoreactive trypsinogen decline in infants with cystic fibrosis identified through newborn screening. J. Pediatr..

[11] Rook J.M., Chervu N., Calkins K.L., Benharash P., DeUgarte D.A. (2025). Meconium-related obstruction and clinical outcomes in term and preterm infants. JAMA Netw. Open.

[12] Munck A., Gérardin M., Alberti C., Ajzenman C., Lebourgeois M., Aigrain Y., Navarro J. (2006). Clinical outcome of cystic fibrosis presenting with or without meconium ileus: A matched ohort study. J. Pediatr. Surg..

[13] Cohen I.S., Moran-Lev H., Levi R., Golden H., Sukhotnik I. (2026). Nutritional strategies for intestinal rehabilitation in children with short bowel syndrome: A narrative review. Nutrients.

[14] Stoop T.F., van Bodegraven E.A., Haaft B.H.E.A.T., van Etten-Jamaludin F.S., van Zundert S.M.C., Lambe C., Tabbers M.M., Gorter R.R. (2024). Systematic review on management of high-output enterostomy in children: An urgent call for evidence. J. Pediatr. Gastroenterol. Nutr..

[15] Solis-Garcia G., Jasani B. (2023). Mucous fistula in neonates: A systematic review and meta-analysis. Arch. Dis. Child Fetal Neonatal Ed..

[16] Elliott T., Walton J.M. (2019). Safety of mucous fistula refeeding in neonates with functional short bowel syndrome: A retrospective review. J. Pediatr. Surg..

[17] Musleh L., Cozzi I., Di Napoli A., Fusaro F. (2025). Mucous fistula refeeding in newborns: Why, when, how, and where? Insights from a systematic review. Nutrients.

[18] Prescribing Information for ZENPEP. https://www.zenpep.com/home-hcp/sites/g/files/lpfasj1066/files/2026-02/Zenpep-USPI-Update_License-change.pdf.

[19] Prescribing Information for CREON. https://www.rxabbvie.com/pdf/creon_pi.pdf.

[20] Prescribing Information for PERTZYE. https://www.pertzye.com/wp-content/uploads/2024/03/24007-Pertzye-PIMGIFU-022024-linked.pdf.

[21] Prescribing Information for PANCREAZE. https://hcp.pancreaze.com/assets/pdf/PANCREAZE-prescribing-information.pdf.

[22] Brownell J.N., Bashaw H., Stallings V. (2019). Growth and nutrition in cystic fibrosis. Semin Respir. Crit. Care Med..

[23] Mountford C.G., Manas D.M., Thompson N.P. (2014). A practical approach to the management of high-output stoma. Frontline Gastroenterol..

[24] Relizorb Compatibility Guide. https://www.relizorb.com/pdf/RELiZORB-Compatible-Formulas-and-Pumps.pdf.

[25] Turck D., Braegger C.P., Colombo C., Declercq D., Morton A., Pancheva R., Robberecht E., Stern M., Strandvik B., Wolfe S. (2016). ESPEN-ESPGHAN-ECFS guidelines on nutrition are for infants, children and adults with cystic fibrosis. Clin. Nutr..

[26] Stallings V.A., Stark L.J. (2008). Evidence-based practice recommendations for nutrition-related management of children and adults in cystic fibrosis and pancreatic insufficiency: Results of systematic review. J. Am. Diet. Assoc..

[27] Choi S., Casey L., Albersheim S., Van Oerle R., Irvine M.A., Piper H.G. (2022). Urine sodium to urine creatinine ratio as a marker of total body sodium in infants with intestinal failure. J. Pediatr. Surg..

[28] Daftary A., Acton J., Heubi J., Amin R. (2006). Fecal elastase-1: Utility in pancreatic function in cystic fibrosis. J. Cyst. Fibros..

